# Quercetin and Thyroid

**DOI:** 10.3390/antiox13101202

**Published:** 2024-10-04

**Authors:** Cesidio Giuliani, Giulia Di Dalmazi, Ines Bucci, Giorgio Napolitano

**Affiliations:** Unit of Endocrinology, Department of Medicine and Sciences of Aging and Center for Advanced Studies and Technology (CAST), University of Chieti-Pescara, 66100 Chieti, Italy; giulia.didalmazi@unich.it (G.D.D.); ines.bucci@unich.it (I.B.); giorgio.napolitano@unich.it (G.N.)

**Keywords:** quercetin, flavonoids, thyroid, endocrine disruptors, FRTL-5 cells

## Abstract

Quercetin is the most abundant flavonoid present in fruits and vegetables. For its antiproliferative, antiviral, anti-inflammatory and antioxidants activities, it is an active ingredient of several herbal remedies and is available as a nutraceutical. Experimental studies performed in vitro have demonstrated that quercetin inhibits growth and function in normal thyroid cells and may act as a thyroid disruptor. These effects have also been confirmed in vivo using rodent models. Some studies have reported the ability of quercetin to interfere with the metabolism of thyroid hormones, since it inhibits the 5′-deiodinase type 1 (D1) activity in the thyroid, as well as in the liver. Besides the effects on normal thyroid cells, several experiments performed in vitro have shown a potential therapeutic role of quercetin in thyroid cancer. Indeed, quercetin inhibits the growth, the adhesion and the migration of thyroid cancer cells, and it also has redifferentiation properties in some thyroid cancer cell lines. In conclusion, these data suggest that, although its effects can be of benefit in hyperthyroidism and thyroid cancer, caution is required in the use of high doses of quercetin due to its anti-thyroid properties. Further in vivo studies are certainly needed to confirm these hypotheses.

## 1. Introduction

**Figure 1:**
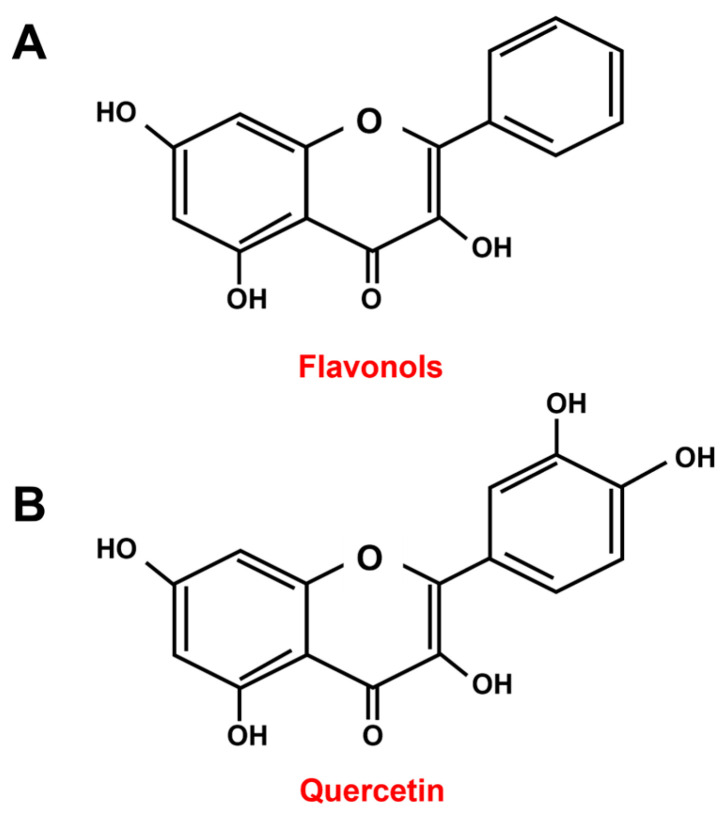
Chemical structures of the flavonols subgroup (**A**) and quercetin (**B**).

Quercetin (3,3′,4′,5,7-pentahydroxyflavone) is a flavonoid belonging to the subgroup of flavonols (Figure 1) [1,2].

It is the most abundant flavonoid present in fruits and vegetables, and it is an active ingredient of several herbal remedies such as *Ginkgo biloba*, *Hypericum perforatum* (St. John’s wort), and Annona squamosa [3,4]. Quercetin has been extensively studied for its antiproliferative, antiviral, anti-inflammatory and antioxidants activities and for its potential effect in cancer and in cardiovascular, inflammatory and infective diseases [5,6,7,8,9,10]. For these properties, quercetin is available worldwide as a nutraceutical with a recommended dose of 100–1200 mg/day, and its intake can reach 2 g/day [11,12]. Several experimental studies have demonstrated an anti-thyroid effect of quercetin that has raised concern about its potential thyroid disruptive activity [12]. However, these data have also spurred several studies to evaluate the potential therapeutic use of this compound in thyroid cancer, given its antiproliferative activity [13,14].

In this review, we summarize the effects of quercetin on normal thyroid function and in thyroid cancer, evaluating their potential clinical impact. 

## 2. Effects of Quercetin on Normal Thyroid Function

The first effect of quercetin on thyroid cells was observed more than 30 years ago in porcine thyroid culture cells, where this compound exhibited a biphasic effect on protein kinase C (PKC) activity (stimulation at lower concentrations and inhibition at higher concentrations) [15]. However, no further information was obtained on thyroid function. A subsequent study showed that the inhibitory effect of quercetin on PKC counteracted the stimulation of iodide organification induced by norepinephrine in thyroid tissues [16]. Afterwards, Divi and Doerge [17], testing the effects of several flavonoids on the activity of thyroid peroxidase (TPO) purified from porcine thyroid, observed a 63.9 ± 12.59% inhibition of the enzyme activity by quercetin. The half-maximal inhibitory concentration (IC_50_) of quercetin was 2.4 ± 0.64 μM, a concentration that can be reached in human plasma of subjects eating high quantities of vegetables or taking dietary supplements of quercetin [12]. The experiments performed suggested a direct interaction between quercetin and TPO that leads to an irreversible inactivation of the enzyme. Further experiments demonstrated that the inhibition of TPO by quercetin is caused by a noncompetitive mechanism due to binding to an allosteric binding site, which modifies the conformation of the enzyme, decreasing its affinity for the substrate [18]. This study has showed a potential anti-thyroid effect of quercetin like that of other flavonoids, although it had the limitation of using purified TPO and not the living cell. 

Based on this, our group performed a series of experiments to evaluate the effects of quercetin on thyroid cell growth and function [4,19]. For this purpose, we used FRTL-5 cells, a non-transformed rat thyroid cell line in continuous culture that represents a reliable model for the study of thyroid cell physiology [19,20,21,22,23,24,25].

**Figure 2:**
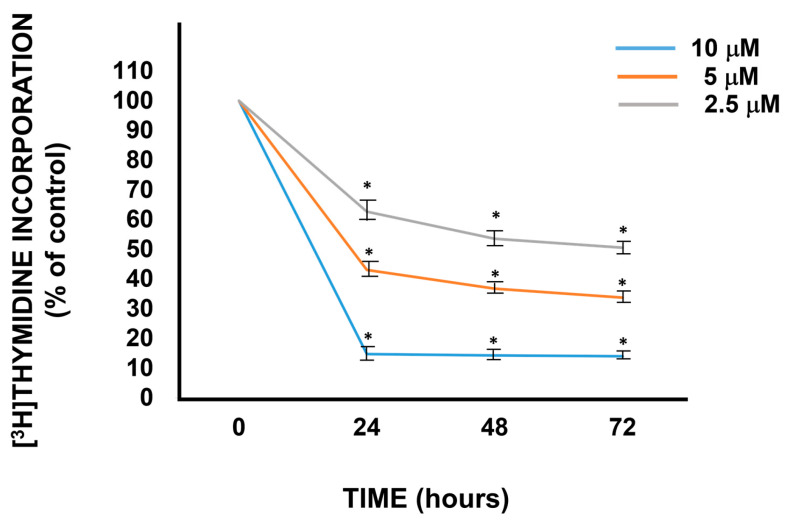
Inhibition of tritiated thymidine incorporation into DNA in FRTL-5 cells by quercetin. Cells were treated with various concentrations of quercetin (2.5 μM, grey line; 5 μM, orange line; 10 μM, light blue line) for the indicated time. Data points represent the mean ± SD of three separate experiments performed in triplicate and are expressed as percentages relative to the control (set to 100%). Comparable data were observed with the cell proliferation assay. * *p* < 0.05. From Reference [18].

Our studies have evinced that quercetin decreased the FRTL-5 cell growth in a dose-dependent manner (Figure 2). The growth inhibition was significant at 2.5 μM and decreased to 16 ± 5% of the control value at 10 μM, with a maximal effect 24 h after treatment [19]. Noteworthy, these concentrations are reached in human plasma after the ingestion of high amounts of vegetables or dietary supplements [12,26,27,28].

A molecular mechanism involved in this antiproliferative effect of quercetin is the inhibition of the phosphatidylinositol 3-kinase (PI3K)/Akt pathway. Although the mechanism by which quercetin exerts its inhibitory effect is still unknown, it is a matter of fact that quercetin inhibits the Akt phosphorylation induced by insulin or serum in the FRTL-5 cells [19]. It is possible that quercetin binds directly to PI3K, inhibiting Akt phosphorylation, as observed in the H-Ras-transformed MCF10 breast cells [29]. We cannot definitely exclude an effect mediated by the antioxidant properties of the compound, as the intracellular excess of reactive oxygen species (ROS) activates several signaling pathways, including PI3K [30,31], and so the radical scavenging effect of quercetin can inhibit this process [31,32]. 

**Figure 3:**
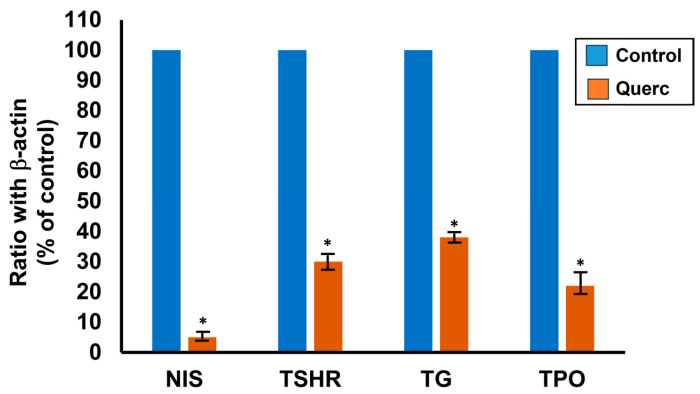
Inhibition of the thyroid-specific genes NIS, TSHR, TG and TPO RNA expression by quercetin 10 μM after 48 h of treatment in the FRTL-5 cells. Data are expressed as percentages relative to the control (set to 100%) and represent the normalized (against actin) mean ± SD of three separate experiments. * *p* < 0.05. Data from References [4,18].

Besides the effect on thyroid growth, quercetin also decreased the expression of the following thyroid-specific genes: sodium/iodide symporter (NIS), thyroglobulin (TG), TPO and TSH receptor (TSHR) [4,19]. The effect was dose- and time-dependent, with a maximal decrease observed with 10 μM after 48 h of treatment (Figure 3). However, a significant reduction was seen in NIS, TSHR and TPO RNA already with 5 μM. These data were confirmed in the expression of the respective proteins and in the iodide uptake [4]. 

An interesting observation from this study was that the inhibitory effect of quercetin on NIS gene expression and iodide uptake involved the phospholipase A_2_ (PLA_2_) pathway and, more specifically, the lipoxygenase pathway [19]. It was observed that the inhibitory effect of quercetin on NIS expression and function was reproduced by treating the cells with ETYA, an inhibitor of the PLA_2_ pathway, and NDGA or Mk-886, inhibitors of the lipoxygenase pathways, whereas the indomethacin, an inhibitor of the cyclooxygenase pathway, did not have any effect [19]. This was a surprising result, since there were no direct data on the involvement of these pathways on NIS gene regulation. 

We do not have further information on the mechanism involved in the effect of quercetin on thyroid gene regulation. We believe that quercetin may act on the expression of the thyroid-specific transcription factors, as has been observed for resveratrol [33]. Indeed, the transcriptional effect of quercetin has also been found in FRTL-5 cells, where it activates AP-1 binding [34]. Of note, the NIS upstream enhancer element (NUE) contains an AP-1-like site, which differs from the canonical site by only one nucleotide (5′-TGACGCA-3′ vs. 5′-TGAC/GTCA-3′) and binds the transcription factors of the AP-1 family, c-jun and c-fos [35]. Although there are no data on the action of quercetin on the NIS promoter, it is important to point out that c-jun activation is negatively correlated with NIS expression [36,37]. 

The inhibitory effect of quercetin on iodide uptake observed in vitro was confirmed in vivo using adult Sprague–Dawley rats [4]. The treatment of rats with quercetin 50 mg/kg/day intraperitoneally for 14 days significantly decreased the radioiodine uptake to 54.12 ± 11% of the control. The dose chosen was equivalent to a dose of about 8 mg/kg in a human, according to dose translation from animal to human [38], and it is known that these high doses are not usually reached with the diet. Indeed, according to the dose translation, a potential anti-thyroid effect could be seen with a chronic ingestion of more than 500 mg/day in an adult. It has been estimated that even a diet rich in fruits and vegetables does not exceed 250 mg/day [11], whereas a dose of 500 mg/day and even more can be reached with the use of quercetin supplements containing 500–1000 mg that are freely available as nutraceuticals [4,12,19]. It must be also considered that the anti-thyroid effect of quercetin can be enhanced by other goitrogenic conditions, such as iodine deficiency or the presence of other thyroid disruptors in food and water. In these conditions, even lower doses of quercetin can impair the thyroid function. Furthermore, we hypothesize that single individuals with a subclinical impairment of thyroid function (i.e., dyshormonogenesis or thyroid autoimmunity) can be sensitive to lower doses of quercetin.

The in vivo anti-thyroid effects of quercetin were also seen in another model for Swiss Albino mice [39]. Here, the treatment of these mice with quercetin 10 mg/kg/day, given orally, decreased the serum concentrations of thyroid hormones both in euthyroid and thyrotoxic animals.

A recent study [40] performed in Sprague–Dawley rats made thyrotoxic by L-T4 administration showed a therapeutic effect of quercetin, which increased the TSH and decreased the T3 and T4 serum concentrations. This effect was dose-dependent, with a significant response at 5 μM and a maximal response at 100 μM. Since these animals had an iatrogenic thyrotoxicosis and not true hyperthyroidism, a possible explanation of these data is that quercetin inhibited the residual thyroid gland activity (TSH is decreased but not suppressed in these thyrotoxic animals).

A further confirmation of the anti-thyroid effects of quercetin is provided by a study showing that long-term treatment of rats (F344/N) and mice (B6C3F1/N) with *Ginkgo Biloba* extracts (in which quercetin is a major ingredient) caused an increase in TSH plasma concentrations with hypertrophy of the thyroid cells [41].

However, we do not have data on the anti-thyroid effects of quercetin in human. In the few clinical trials performed, no effects were reported regarding thyroid growth and function [11,42]. Of note, although the dose of quercetin administered in several trials is ≥500 mg/day, i.e., comparable to that used in rats [4], the length of treatment has been no longer than 12 weeks. This time is significantly shorter than the treatment performed in the rats, which corresponded to at least 52 weeks, according to the human–rodent translation time [43]. It should be also highlighted that, in these clinical trials, the thyroid function was not evaluated, avoiding the detection of possible subclinical abnormalities.

## 3. Effects of Quercetin on Thyroid Hormone Metabolism

Besides the effects on thyroid cells, quercetin also acts on thyroid hormone metabolism, inhibiting the enzyme deiodinases.

Studies performed in vitro have shown that quercetin inhibits intrathyroidal 5′-deiodinase type 1 (D1), with an IC_50_ of 13.23 ± 1.51 μM [44]. This effect was also observed in vivo in Swiss Albino mice treated with quercetin 10 mg/kg/day orally, where an inhibition of the liver D1 activity was seen in euthyroid and thyrotoxic animals [39]. D1 is the enzyme responsible for the conversion of T_4_ in T_3_, as the latter constitutes the biologically active hormone. This effect further contributes to the anti-thyroid activity of quercetin decreasing the serum concentrations of T_3_. The inhibitory effect of quercetin on D1 can be mediated by the antioxidant properties of the compound [40].

These data further confirm a role of quercetin as a thyroid disruptor.

## 4. Effects of Quercetin on Thyroid Cancer Cells

The antiproliferative effect of several flavonoids on thyroid cancer cells has been known for over 20 years [45]. A seminal study performed in 2011 showed that quercetin also had an inhibitory effect on the growth of several types of thyroid cancer cell lines: TPC-1 (a papillary thyroid cancer cell line), FTC-133 (a follicular thyroid cancer cell line), NPA (a poorly differentiated papillary thyroid cancer cell line), FRO and ARO (anaplastic thyroid cancer cell lines) [46]. In detail, the treatment of the aforementioned cell lines with quercetin for 72 h inhibited cell growth in a dose-dependent manner. A small but significant effect was found at a quercetin concentration of 10 μM in all the cell lines, except TPC-1, whereas an inhibitory effect greater than 40% was seen in all the cell lines with a dose of 50–100 μM. These concentrations can easily be obtained in humans by the intravenous administration of quercetin [47,48]. Quercetin treatment also decreased the expression of the CD97 gene in FTC-133, NPA, FRO and ARO cells. This gene is considered a marker of dedifferentiation and induces cell migration and invasion [49]. Furthermore, quercetin showed redifferentiation properties in NPA, FRO and FTC-133 cells, inducing the expression of the NIS gene [46] and the consequent uptake of radioiodine, suggesting its role as adjuvant in radioiodine therapy. These data contrast with that observed in normal thyroid cells, where, as reported above, quercetin downregulates NIS expression and iodide uptake. Of note, this discrepancy can also be found in the treatment of thyroid cells with resveratrol [33], and for this reason, several hypotheses have been formulated to explain this discrepancy. One possibility is represented by a different mechanism of action of the compound based on the cell model used, i.e., normal or neoplastic thyroid cells. Indeed, quercetin, as well as other polyphenols, can have contradictory effects on different in vitro and in vivo models and even on the same cell line based on the cellular functional status [50,51,52,53,54]. Moreover, quercetin shows different effects based on its concentration (hormetic effect) and chemical modifications [55,56,57]. These considerations highlight the need for further studies to understand the mechanism of action of quercetin in normal and cancer thyroid cells.

As already reported, the mechanism of action of quercetin on thyroid cells in not completely understood. Some effects are consequent to a direct binding to enzymes, as seen for TPO [18]; other effects may be linked to the antioxidant properties of quercetin, particularly in its activity as a ROS scavenger [31]. We finally hypothesize that quercetin can also act through the aryl hydrocarbon receptor (AHR), and actually, it is noticed that the AHR mediates the effects of quercetin in other cells [58,59,60]. The possible involvement of AHR might explain the opposite effects of quercetin on the different cells, since AHR signaling is very complex and shows paradoxical effects [61,62,63].

Subsequent studies reported the inhibitory effect of quercetin on the growth of the poorly differentiated papillary thyroid carcinoma cell line B-CPAP. The antiproliferative effect of quercetin on B-CPAP cells was maximal at concentrations between 50 and 100 μM and was linked to the induction of apoptosis by caspase-3 activation [64,65,66,67]. The apoptotic effect is related with the downregulation of the heat shock protein (Hsp) 90 expression [65]. Quercetin was also able to decrease cell adhesion and migration by modifying some markers involved in epithelial–mesenchymal transition (EMT). In detail, an increase in the expression of E-cadherin and a decrease in the expression of N-cadherin and matrix metalloproteinase-9 were reported [66]. Furthermore, it decreased the production of some cytokines involved in tumor progression and survival: IL-4, IL-8, IL-10, IL-1α and TNF-α [64]. The effects on cytokines production and growth were also observed in a medullary thyroid cancer cell line, the TT cells [64]. 

Further studies confirmed the antiproliferative effect of quercetin in other cell lines: the differentiated papillary thyroid cancer cell line K1, the anaplastic thyroid cancer cell line 8505c and the metastatic papillary thyroid cancer cell line MDA-T85 [67]. In detail, quercetin 20 μM induced apoptosis with a decrease in cell proliferation >50% of the control. Additionally, it enhanced the effects of bromodomain and extraterminal domain (BET) inhibitors on cell apoptosis and the sphere-forming ability. The study also showed that the apoptotic effect of quercetin was mediated in part by the inhibition of the heterogeneous nucleoprotein A1 (hnRNPA1) expression. This is a nuclear RNA binding protein involved in the regulation of RNA processing, splicing, export and translation, which is overexpressed in several cancers, contributing to tumor progression [68].

A recent study has proven a synergic effect of quercetin and sorafenib on thyroid cancer cells. Specifically, cotreatment of B-CPAP and K1 cell lines with quercetin 25 μM and sorafenib 0.1 μM had a significant decrease in the cellular growth, migration and adhesion compared to each treatment alone [69].

Table 1 summarizes the main effects of quercetin on thyroid cancer cell lines.

Despite the several studies mentioned above that evaluated the effects of quercetin in thyroid cancer cells, there are no data regarding the effect on differentiated thyroid cancer in vivo and only one study regarding the medullary thyroid cancer. This study has shown that the anti-tumor effect of hyperthermia in athymic nude mice bearing TT cells is dramatically increased by cotreatment with lipopolysaccharide (LPS) and quercetin [70].

## 5. Conclusions

The data revised above indicate two important roles of quercetin on the thyroid: a role as a thyroid disruptor and a role as a potential therapeutic agent in thyroid cancer.

The role of quercetin as a thyroid disruptor is suggested from experimental studies performed both in vitro and in vivo. 

In conclusion, we believe that, although the anti-thyroid effects of quercetin can be beneficial in hyperthyroid patients, as suggested by the studies performed on animal models [39,40,71], as there are no solid data on the effects of quercetin on human thyroids, caution should be taken in the use of quercetin supplements in people at risk for thyroid dysfunction, in pregnant women and in patients who are candidates for radioiodine administration.

Regarding the role of quercetin as an antineoplastic agent in thyroid cancer, it should be underlined that the data available are from studies performed in vitro. Therefore, in vivo studies are mandatory to pursue this hypothesis.

## Figures and Tables

**Table 1:** Main effects of quercetin on thyroid cancer cell lines.

Type of Thyroid Cancer	Cell Lines	Effects	References
Differentiated papillary	TPC-1	Growth inhibition	[46]
	K1	Growth inhibition Decrease of hnRNPA1 protein Increase the effects of bromodomain and extraterminal domain inhibitors on cell apoptosis and sphere-forming ability Synergic effect with sorafenib on growth and EMT inhibition	[67,69]
Differentiated follicular	FTC-133	Growth inhibition Decrease of CD97 expression Induction of NIS expression	[46]
Poorly differentiated papillary	NPA	Growth inhibition Decrease of CD97 expression Induction of NIS expression	[46]
	B-CPAP	Growth inhibition Decrease of cytokines production Apoptosis Decrease of Hsp90 expression Decrease of cell adhesion and migration Decrease of N-cadherin expression Decrease of MMP-9 expression Increase of E-cadherin expression Increase of NIS expression and radio-iodine uptake Synergic effect with sorafenib on growth and EMT inhibition	[64,65,66,69]
Anaplastic	ARO	Growth inhibition Decrease of CD97 expression	[46]
	FRO	Growth inhibition Decrease of CD97 expression Induction of NIS expression	[46]
	8505c	Growth inhibition Decrease of hnRNPA1 protein levels Increase the effects of bromodomain and extraterminal domain inhibitors on cell apoptosis and sphere-forming ability	[67]
Metastatic papillary	MDA-T85	Growth inhibition Decrease of hnRNPA1 protein levels Increase the effects of bromodomain and extraterminal domain inhibitors on cell apoptosis and sphere-forming ability	[67]
Medullary	TT	Growth inhibition Decrease of cytokines production	[64]

## Data Availability

The data presented in this review are available in the references cited.
